# Prevalence of Mild Cognitive Impairment in Latin America and the Caribbean: A Systematic Review

**DOI:** 10.1093/geroni/igaa057.3307

**Published:** 2020-12-16

**Authors:** Fabiana Ribeiro, Ana Carolina Teixeira-Santos, Anja Leist

**Affiliations:** 1 University of Luxembourg, Esch-sur-Alzette, Luxembourg; 2 University of Minho, Braga, Portugal

## Abstract

Background. The population of Latin America and the Caribbean (LAC) is ageing rapidly, presenting the highest prevalence rates of dementia in the world. In this context, mild cognitive impairment (MCI) is an intermediate condition between normal ageing and dementia. However, very few studies verified the prevalence of MCI in LAC countries; earlier global systematic reviews only considered prevalence reports published in English language. Method. We conducted a systematic review to evaluate the prevalence of MCI in LAC countries and to explore the factors associated with MCI (i.e., age, gender, and education). A database search was conducted in February 2020 using PubMed, Web of Science, Scopus, Lilacs, SciELO, and EMBASE, for population-or community-based studies with MCI data for countries in LAC, published in English, Spanish, or Portuguese language. From k=2,168 identified and k=1,684 screened studies, only articles were selected that included subjects with a precise diagnosis of MCI. The studies were qualitatively assessed using the JBI critical appraisal checklist for studies reporting prevalence data tool. Results. A total of nine studies met the criteria, published between 2007 and 2019, including a total of 17,812 participants in nine countries Brazil, Mexico, Argentina, Colombia, Peru, Cuba, Dominican Republic, Venezuela, and Costa Rica. Estimates for MCI prevalence ranged from 1.2% to 34%, with most estimates between 1.2% and 6.45%. Estimates differed by age group, gender, and educational level. Discussion. This is the first systematic review of the prevalence of MCI in LAC countries, considering only high-quality studies adopting rigorous diagnostic criteria.

